# Self-Medication of ADHD Symptoms: Does Caffeine Have a Role?

**DOI:** 10.3389/fpsyt.2022.813545

**Published:** 2022-02-03

**Authors:** Csilla Ágoston, Róbert Urbán, Zsolt Horváth, Wim van den Brink, Zsolt Demetrovics

**Affiliations:** ^1^Institute of People-Environment Transaction, ELTE Eötvös Loránd University, Budapest, Hungary; ^2^Institute of Psychology, ELTE Eötvös Loránd University, Budapest, Hungary; ^3^Amsterdam University Medical Centers, Location Academic Medical Center, University of Amsterdam, Amsterdam, Netherlands; ^4^Centre of Excellence in Responsible Gaming, University of Gibraltar, Gibraltar, Gibraltar

**Keywords:** caffeine, caffeine use disorder, ADHD, well-being, self-medication

## Abstract

**Objective:**

Stimulants are the most effective treatment for Attention Deficit/ Hyperactivity disorder (ADHD). In addition, studies have shown that nicotine dependence in patients with ADHD is probably best explained by self-medication. The question is whether this is also true for caffeine use and caffeine dependence. The aim of our study was, therefore, to examine the relationship of ADHD symptoms, caffeine consumption, caffeine use disorder (CUD) and well-being. We hypothesized that those who have more ADHD symptoms and regularly consume caffeine have higher psychological well-being than those who have more ADHD symptoms, but do not consume caffeine.

**Methods:**

A general population sample (*N* = 2,259, 70.5% male, mean age 34.0) filled out the 10-item Caffeine Use Disorder Questionnaire (CUDQ), the Adult ADHD Self-report Scale (ASRS) and the WHO-5 Well-Being Index (WHO-5) and were asked about their caffeine consumption habits in an online survey.

**Results:**

There were no associations between ADHD and coffee, tea, energy drink or cola consumption or daily caffeine consumption. However, the results of the path analysis showed that the level of ADHD symptoms was positively associated with the level of CUD (β = 0.350) and negatively with the WHO-5 (β = −0.259).

**Conclusions:**

Caffeine consumption was not associated with ADHD symptom severity and thus not likely to represent self-medication. On the contrary, caffeine use disorder severity is associated with more ADHD symptoms and both caffeine use disorder and ADHD are associated with lower well-being.

## Introduction

Attention-deficit/hyperactivity disorder (ADHD) can be characterized by a pattern of attention deficit, hyperactivity and/or impulsivity, which interferes with development or daily functioning (1). The prevalence of ADHD in the general population is worldwide about 6% in childhood/ adolescence and about 2.5–7.2% in adulthood (2–5). Children and adults with ADHD have lower quality of life and lower subjective well-being compared with children and adults without ADHD (6, 7).

Stimulant medication (e.g., dextroamphetamine, methylphenidate) is an evidence based and accepted treatment option for ADHD (8, 9). Therefore, the question arises whether other–relatively mild–stimulants, such as nicotine and caffeine could also alleviate ADHD symptoms, and thus be used as some kind of self-medication (9–11). Indeed, a recent review concluded that nicotine dependence in patients with ADHD is probably best explained by self-medication (12). The question is whether this is also true for caffeine use and caffeine dependence.

The effects of caffeine on ADHD symptoms have been studied in several animal studies, comparing spontaneous hypertensive (SHR) rats with Wistar (WIS) rats (13, 14) or Wistar Kyoto (WKY) rats (15), or using 6-OHDA lesioned rats (16). According to Prediger et al. (13), 1 to 10 mg/kg pre-training administration of caffeine improved the spatial learning deficit in SHR rats, but did not alter the performance of WIS rats. Pires et al. (14) found that both long-term caffeine (3 mg/kg) and methylphenidate treatment (2 mg/kg) in prepubertal age improved the deficits in object-recognition in SHR rats (however, both treatment deteriorated object-recognition in WIS rats). In the experiment of Pandolfo et al. (15), chronic caffeine treatment (2 mg/kg) did not affect the performance of WKY rats while it improved memory deficits as well as inattention in SHR rats. Caballero et al. (16) found that long-term caffeine treatment in prepubertal age did not alter motor activity in either 6-OHDA lesioned rats or saline-treated rats, but it improved the attention deficit of the 6-OHDA lesioned rats. Overall, animal experiments suggest that certain symptoms of ADHD (spatial learning deficits, memory problems, attention deficit) are improved by caffeine, whereas caffeine has generally no effect on non-ADHD like rats.

A review of studies from the 1970–80s (17) found that only a relatively small number of studies examined the effectiveness of caffeine in the treatment of ADHD (or minimal brain dysfunction) and these studies usually had small sample sizes or weak protocols. Most of these studies have found that caffeine is less effective than methylphenidate and d-amphetamine, but it was beneficial for some participants. Stein et al. (18) conducted a meta-analysis of 21 studies examining the effects of theophylline and caffeine on children's cognition and found that both methylxanthines slightly reduced children's externalizing behavior (e.g., hyperactivity, problematic or aggressive behavior) based on parents' evaluation. Another review (19) focused especially on those studies, which examined caffeine's effects on the cognitive, psychomotor or affective functioning of children with ADHD. This review concluded that caffeine was more effective than no treatment or placebo for ADHD severity, executive functions, hyperactivity, impulsivity and aggression according to parents and teachers. However, methylphenidate and amphetamines were more effective than caffeine for these indicators. The combination of caffeine and other stimulants (if they eventuate a moderate increase in arousal) may lead to better results than the separate use of each compound (19). Although these results were applied only to children, Liu et al. (9) argue that it would be worth examining the efficacy of tea consumption for the treatment of adult ADHD because it is likely to be a suitable form of treatment for those who are difficult to involve in other medication treatments. Ioannidis, Chamberlain and Müller (20) chronologically reviewed those studies related to ADHD and caffeine and pointed out that caffeine may be mistakenly excluded from the repertoire of ADHD medications and it could be especially useful for the treatment of mild/moderate adult ADHD. According to Ross and Ross (1982, cited by (21)) the ideal therapeutic dose of caffeine would be 100–150 mg for children (which is equivalent to about 1–2 cups of coffee), but adults may need higher doses. It is also important to consider the possible consequences of long-term caffeine treatment such as tolerance (20). Drawing the right conclusions may be hampered by the methodological differences of the studies. Therefore, Grimes et al. (22) examined the methodological background of 16 experiments that focused on the effects of caffeine on ADHD and found that the experiments showed a high degree of variability in sampling, the dose of caffeine used in the experiment, the duration of treatment, the design of the experiment, and the dependent variables (e. g. physiological measures, performance tests, etc.). The possible benefits of using caffeine compared to other stimulants in the treatment of ADHD would be that it is easily available and has low addictive potential (15, 17) and its use is less stigmatized compared to other substances (20).

General caffeine consumption patterns and ADHD have been investigated in cross-sectional studies with adults (mainly students) and children. Martin et al. (23) found that in adolescents high caffeine consumption is associated with a variety of externalizing behaviors (e.g., aggressive behavior, ADHD). Caffeine consumption among smokers is associated with a higher number of ADHD symptoms, depression and anxiety among young adults (24). Kelly and Prichard (25) have studied risk behavior, sleep patterns and mental disorders among university students, comparing frequent energy drink consumers (3 or more cans/month) and frequent coffee consumers (16 or more coffee/month) with those who consume less energy drinks/coffee. They found that frequent energy drinkers reported more risk behavior (e.g., increased alcohol and drug use) and sleep problems and more often had a mental disorder (including ADHD) compared to those who consumed less energy drinks, while there were no differences between more frequent and less frequent coffee consumers. According to Walker et al. (26), adolescents with ADHD diagnosis are twice as likely to consume caffeinated drinks (coffee and/or other caffeinated drinks) than those without ADHD. Cipollone et al. (27) found similar patterns among soldiers: those with ADHD diagnosis tended to consume more caffeinated products and also had a higher prevalence of SUD than those without an ADHD diagnosis. However, not only the consumption of traditional caffeinated drinks (e. g. coffee, soft drinks, energy drinks) has been associated with ADHD. The results of Van Eck, Markle and Flory (28) suggest that ADHD symptoms also predict the consumption of caffeine-containing over-the-counter medications. Although caffeine consumption has been associated with several positive health effects (29), regular consumers can develop a problematic pattern of caffeine use. Caffeine withdrawal has been included in DSM-5, while caffeine use disorder (CUD) has been listed as a “condition for further study” (1).

In general, these results suggest that habitual caffeine consumption has a positive correlation with the presence of ADHD symptoms/diagnosis. It is important to note that research in this field is extremely heterogeneous regarding the age of participants as well as the measurement of ADHD and caffeine consumption. The majority of studies did not separate the consumption of various caffeinated products and there were also differences in the main focus of the studies: some of them focused mainly on caffeine consumption, and ADHD was a more peripheral topic [e.g., 25], while in other studies ADHD was the main focus instead of caffeine consumption [e.g., 28]. It is also important to note that, due to its effects on different neurotransmitter systems, caffeine can have both beneficial and detrimental effects not only on ADHD, but on several other mental disorders as well, such as mood disorders, anxiety disorders and schizophrenia, and besides the possible self-medication motives, some patients may consume it to counteract the side effects of their medication (17). The frequent comorbidity of mental disorders (30, 31) also challenges a better understanding of the effects of caffeine. Although measurement tools developed to screen for adult ADHD–such as the ADHD Self-Report Scale-V1.1 (ASRS-V1.1), which was used in the current study–are reliable and have good convergent and divergent validity (32, 33), the identification of adult ADHD is not always straightforward: some symptoms may be obscured by the consequences of a chronic illness, such as substance use disorders, and some symptoms may overlap with those of affective disorders (34). Therefore, the results of studies using a cross-sectional design and screening tools to establish the presence of probable disorders should be interpreted from a transnosographic or transdiagnostic perspective.

The possibility that caffeine is used by people with ADHD– or ADHD-like symptoms–as a kind of self-medication strategy has been raised by several authors (17, 19, 20), but so far the complex relationship between ADHD symptoms, the consumption of different caffeinated beverages, caffeine use disorder (CUD), and psychological well-being has not been studied. Including CUD symptoms and psychological well-being, as variables, allows us to explore the mediating effect of caffeine consumption between ADHD symptoms and well-being: can people successfully compensate the symptoms of ADHD by using caffeine or do they rather experience the negative consequences of caffeine consumption? Therefore, we hypothesize that those who have more ADHD symptoms and regularly consume caffeine have higher psychological well-being than those who have more ADHD symptoms but do not consume caffeine.

## Materials and Methods

### Sample and Procedures

A sample from the adult general population (*N* = 2,259) was asked about its caffeine consumption habits, ADHD symptoms and well-being in a cross-sectional online survey using convenience sampling. The questionnaire was presented on one of the biggest and most visited news website of Hungary (www.444.hu) and adults (above 18 years) who consume caffeine at least weekly were invited to participate.

The study was approved by the Research Ethics Committee of ELTE Faculty of Education and Psychology. The number of the ethical approval is 2015/254. Participants could read the informed consent after they clicked on the hyperlink of the questionnaire and they could carry on with the questionnaire only if they marked in a check box that they read the consent.

### Measures

#### Caffeine Consumption

Participants reported the frequency and quantity of coffee, instant coffee, tea (black and green), energy drink, cola and caffeine pill consumption on an eight-point scale (0 = never, 1 = weekly or less, 2 = several times a week, 3 = one portion per day, 4 = two portions per day, 5 = three portions per day, 6 = four portions per day, 7 = five or more portions per day). Total daily caffeine consumption was computed from the daily use of different caffeinated beverages. The consumption of coffee, tea, energy drink and cola was dichotomized (consumes it daily or not). The method of calculation of caffeine content was published elsewhere (35).

#### Caffeine Use Disorder Symptoms

Participants filled out the 10-item Caffeine Use Disorder Questionnaire (CUDQ), which aims to assess the presence of caffeine use disorder symptoms during the last 12 months. The answers are scored on a four-point Likert scale (1 = never, 2= sometimes, 3 = often, 4 = very often), but the items were transformed into binary answers by combing the last three answering options into one “yes” answer. The discriminative value and severity of the various items of CUDQ were reported in another article (36). Internal consistency of the CUDQ total score was acceptable in the current study (α = 0.71).

#### ADHD Symptoms

We used the Adult ADHD Self-Report Scale-V1.1 (ASRS-V1.1) Part A (37) for the assessment of ADHD symptoms. The questionnaire consists of six items which target certain symptoms associated with attention deficit and hyperactivity in the last 6 months. Participants could respond on a five-point Likert-scale and could receive a score of 0–4 for each item. The scale can be used as a continuous variable (Cronbach's alpha = 0.63–0.72) and people can be classified in four groups based on the total score: low negative (0–9 points), high negative (10–13 points), low positive (14–17 points) and high positive (18–24 points) (38). The low negative and high negative groups are more likely to be non-ADHD participants, while the low positive and high positive groups are considered to have ADHD, based on clinical interviews, but there are some differences between the two “negative” and two “positive” categories as well (38, 39). Participants were asked about the age of onset of the symptoms as well. The ASRS-V1.1 had acceptable internal consistency in a previous study with Hungarian adults (α = 0.72) (40) and in the current study (α = 0.75).

#### Well-Being

The five-item WHO Well-Being Index (WHO-5) was used for the evaluation of psychological well-being. The one-factor WHO-5 had excellent internal consistency in a previous study on a representative sample in Hungary (α = 0.85) (41) and in the current study (α = 0.80).

### Statistical Analysis

ADHD symptoms were used as an independent categorical variable in the Chi-square tests and ANOVAs. The ADHD categories were based on the recommendations of Kessler et al. (38) (see in the Section Measures).

The probability of the consumption of each caffeinated beverage (coffee/tea/energy drink/cola) in the four ADHD groups was compared by using Chi-square tests. We also compared the four ADHD groups regarding the magnitude of daily caffeine consumption and caffeine use disorder symptoms by using ANOVAs with Games-Howell *post hoc* tests. The bivariate associations of the variables were examined by Pearson correlations.

Two path models with observed variables were used to explore the relationship between self-reported ADHD symptoms, caffeine consumption, caffeine use disorder symptoms and psychological well-being. We used the total score on the ASRS-V1.1 as a continuous independent variable, the score on WHO-5 as a continuous dependent variable and the CUDQ total score as a continuous mediator variable in both models. We used the total daily caffeine consumption as a continuous mediator variable in the first model and coffee, tea, cola and energy drink consumption as dichotomous mediator variables (consumes it daily/does not consume it daily) in the second model. In the first model, we used the maximum likelihood estimation method (ML) because all variables were continuous. In the second path model we used probit regression with WLSMV estimation and delta parameterization because of the dichotomous mediator variables. We used the STDYX output of Mplus to determine the standardized regression coefficients (β) and the indirect effects.

Model fit was investigated by examining χ^2^-test statistic, the Comparative Fit Index (CFI) (acceptable above 0.90, excellent above 0.95) (42–44), the Tucker–Lewis Index (TLI) (acceptable above 0.90, excellent above 0.95) (43–45), the Root Mean Square Error of Approximation (RMSEA) (acceptable below 0.08, excellent below 0.05) (43, 44, 46) and the 90% confidence interval of RMSEA (47).

The two path analyses were performed with MPLUS 6.0 (48) and the descriptive statistics, Cronbach's alphas, Chi-square tests, Pearson correlations and ANOVAs were performed with SPSS 22 (49).

## Results

### Sample Characteristics

Most participants (70.5%) were male, and the mean age was 34.0 years (SD = 9.3 years). This is a generally well-educated group with 73.5% having a college degree or higher, 24.9% with a high school diploma, and only 1.6% with elementary school or vocational school as the highest educational attainment. Most of the participants were employed with 77.5% having a full-time job, 10.2% having less than full-time job and “only” 12.3% being unemployed. The sample was mainly urban with 63.1% of the participants living in Budapest, 31.1% living in other cities, and only 5.8% living in a town or village.

Almost all participants (92.1%) were daily caffeine users. The average daily caffeine consumption was 255.40 mg (SD = 145.36) for males (which is the equivalent to about 2.5 cups of coffee) and 223.35 mg (SD = 125.61) for females (which is the equivalent to about 2.3 cups of coffee), which is higher than the average consumption: 121.70 mg/day for males (1.2 cups/day) and 123.1 mg/day for females (1.2 cups/day) in Hungary in 2009 (50). Participants reported a mean of 3.11 (SD = 2.04) CUD symptoms in the last year.

The mean score for the WHO-5 was 8.46 (SD = 2.86) and for the ASRS-V1.1. 8.21 (SD = 4.48). The average age of ADHD symptom detection was 16.8 years (SD = 10.4). Of all 2,259 participants, 59.8% (*n* = 1,351) belonged to the low negative category, 25.8% (*n* = 583) to the high negative category, 9.3% (*n* = 210) to the low positive category, and 2.3% (*n* = 52) to the high positive category (missing data: 2.8%, *n* = 63).

### ADHD, Caffeine Consumption and CUD

There were no significant differences between the four ADHD groups in daily coffee consumption [$\chi_{\left(3\right)}^{2}$ = 0.722, *p* = 0.868], tea consumption [$\chi_{\left(3\right)}^{2}$ = 6.674, *p* = 0.083], cola consumption [$\chi_{\left(3\right)}^{2}$ = 1.989, *p* = 0.575] and energy drink consumption [$\chi_{\left(3\right)}^{2}$ = 0.942, *p* = 0.815].

**Figure 1:**
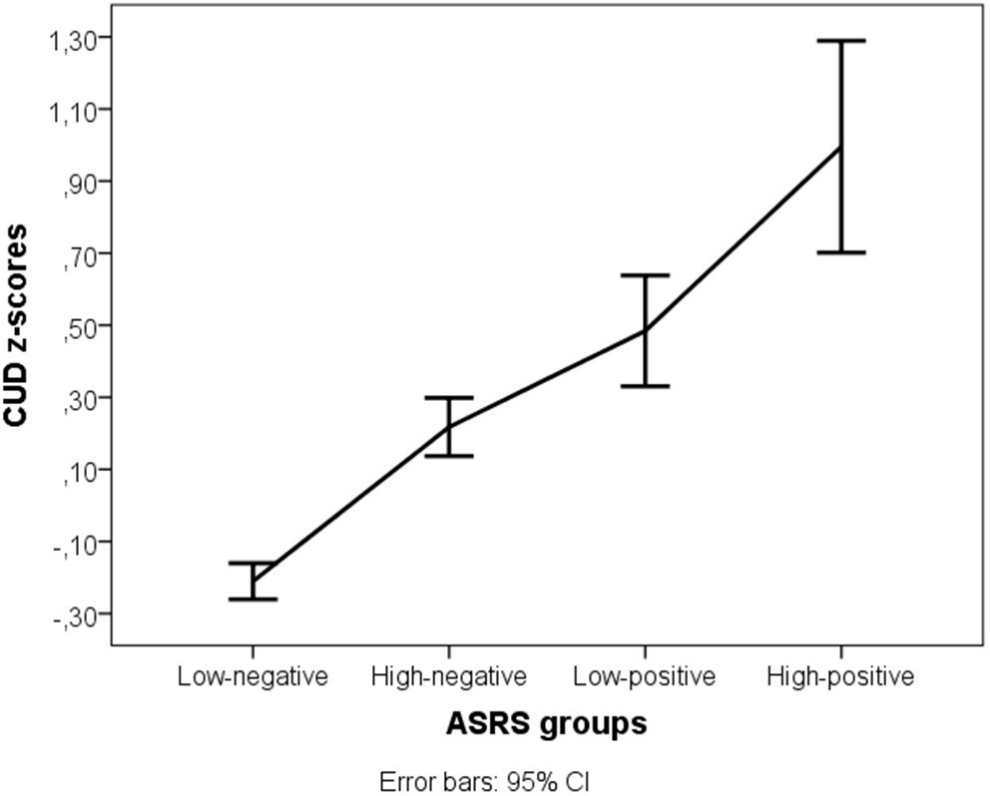
Caffeine use disorder symptom z-scores in the four ADHD groups. ASRS, Attention-deficit/hyperactivity disorder symptoms; CUD, Caffeine Use Disorder symptoms.

Daily caffeine consumption and the CUDQ score were normally distributed, however the requirement of homogeneity of variances was fulfilled only for daily caffeine consumption [F_(3,2,191)_ = 0.276, *p* = 0.843] but not for CUDQ scores [F_(3,2,092)_ = 4.765, *p* = 0.003]. There was no difference between the four ADHD groups in daily caffeine consumption [F_(3)_ = 0.823, *p* = 0.481], but the groups had significantly different CUDQ scores [Welch F_(3,202.001)_ = 59.207, *p* < 0.001, *r* = 0.29]. The *post-hoc* test showed that each group significantly differed from the others: the low negative ADHD group had the lowest CUDQ score compared to the other three ADHD groups (*p* < 0.001), the high negative ADHD group significantly differed from the low positive (*p* = 0.014) and high positive ADHD groups (*p* < 0.001), and the low positive ADHD group also differed from the high positive ADHD group (*p* = 0.015) (Figure 1).

### Correlations Between ADHD Symptoms, Caffeine Consumption Variables, CUD Symptoms, and Well-Being

The correlations of the variables are presented in Table 1. The number of ADHD symptoms was negatively associated with well-being and positively associated with the number of CUD symptoms, while well-being had a moderate negative correlation with CUD symptoms. Neither total daily caffeine consumption, nor the daily consumption of each caffeinated beverage was associated with ADHD symptoms. Interestingly, well-being had small, negative correlations with daily cola and energy drink use and a small positive correlation with daily tea consumption.

**Table 1:** Correlation matrix of the variables in the present study.

	**ADHD**	**well-being**	**total caffeine**	**coffee**	**tea**	**energy drink**	**cola**
well-being	**−0.301**						
total caffeine	0.038	**–**0.029					
coffee^a^	0.041	0.015	**0.574**				
tea^a^	**–**0.036	**0.073**	**0.104**	**−0.104**			
energy drink^a^	0.015	**−0.079**	**0.116**	**−0.113**	**–**0.005		
cola^a^	**–**0.015	**−0.049**	**0.066**	**−0.100**	**–**0.033	**−0.189**	
CUD	**0.357**	**−0.207**	**0.233**	**0.209**	**−0.63**	**0.132**	**0.045**

*N = 2169–1994. Significant correlations (p < 0.05) are boldfaced. ADHD, Attention-deficit/hyperactivity disorder symptoms; CUD, Caffeine Use Disorder symptoms*.

a *Coded as: 0 = Absence of daily consumption, 1 = Presence of daily consumption*.

### Path Models

**Figure 2:**
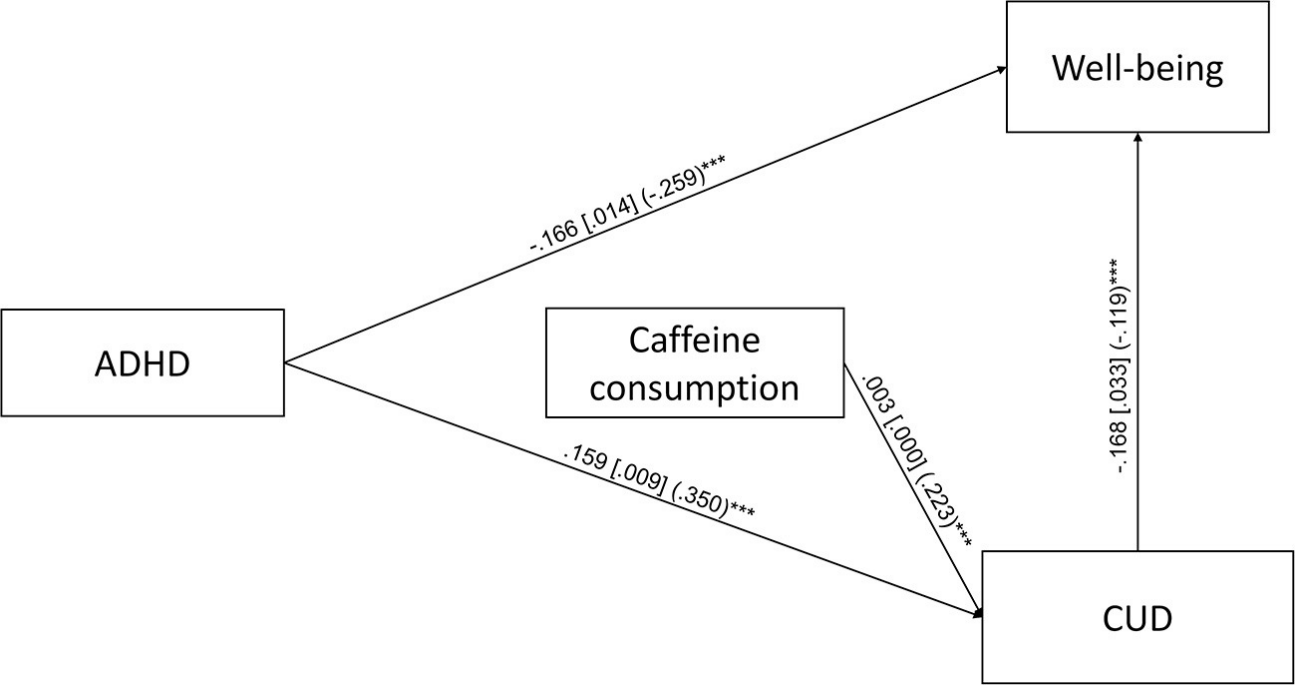
Path analysis for the association of ADHD symptoms, caffeine consumption, caffeine use disorder symptoms (CUD) and well-being (*N* = 2,196). Unstandardized regression coefficients, their standard errors (in brackets) and standardized coefficients (in parentheses) are presented in the figure. Only the significant (*p* < 0.05) direct paths are presented ^***^p < 0.001.

The first path analysis, which included ADHD symptoms, total caffeine consumption, and caffeine use disorder symptoms resulted in a saturated model. The unstandardized and standardized regression coefficients of the first path analysis are depicted on Figure 2. ADHD symptoms and caffeine consumption were positively associated with CUD symptoms, while ADHD symptoms and CUD symptoms were negatively associated with well-being. Caffeine consumption was neither associated with ADHD symptoms, nor with well-being directly. We found two significant indirect paths in the first path analysis: (1) *ADHD* → *CUD* → *well-being* (B = −0.027, S.E. = 0.005, *p* < 0.001, β = −0.042, S.E. = 0.008, *p* < 0.001, total indirect effect from ADHD to well-being: B = −0.026, S.E. = 0.005, *p* < 0.001, β = −0.041, S.E. = 0.008, *p* < 0.001), where more ADHD symptoms predict more CUD symptoms and more CUD symptoms predict lower well-being, and (2) *total caffeine consumption* → *CUD* → *well-being* (*B* = −0.001, S.E. = 0.000, *p* < 0.001, β = −0.026, S.E. = 0.006, *p* < 0.001), where higher total daily caffeine consumption predicts more CUD symptoms and more CUD symptoms predict lower well-being.

**Figure 3:**
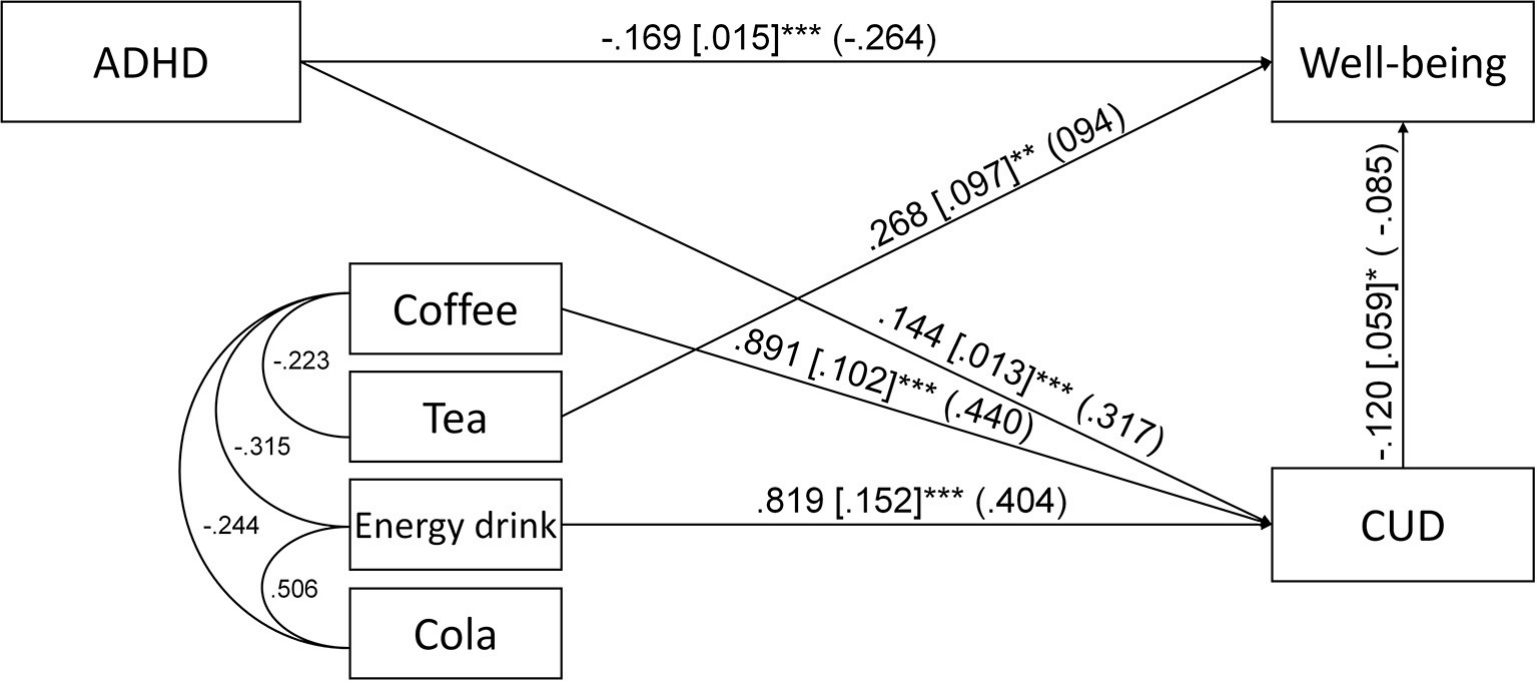
Path analysis for the association of ADHD symptoms, coffee, tea, energy drink and cola consumption, caffeine use disorder symptoms (CUD) and well-being. Unstandardized regression coefficients, their standard errors (in brackets) and standardized coefficients (in parentheses) are presented in the figure. Only the significant (*p* < 0.05) direct paths and error covariances are presented ^*^p < 0.05; ^**^p < 0.01; ^***^p < 0.001.

The second path analysis, which included ADHD symptoms, coffee, tea, cola and energy drink consumption, CUD symptoms and well-being had poor fit indices [χ^2^ = 122.246, df = 6, *p* < 0.001; CFI = 0.854; TLI = 0.488; RMSEA = 0.094 (CI: 0.080–0.109)]. Based on the examination of the modification indices, the covariances between the four caffeinated beverages were introduced to the model. This modified path analysis was a saturated model. The unstandardized and standardized regression coefficients of the second path analysis are depicted on Figure 3. Importantly, none of the caffeinated beverages was associated with ADHD symptoms and only tea consumption was associated with well-being. Coffee and energy drink consumption was associated with more CUD symptoms. For the second path analysis, we found three significant indirect paths: (1) *ADHD* → *CUD* → *well-being* (*B* = −0.017, S.E. = 0.009, *p* = 0.046, β = −0.027, total indirect effect from ADHD to well-being: *B* = −0.022, S.E. = 0.008, *p* = 0.004, β = −0.034), which also appeared in the first path model, (2) c*offee* → *CUD* → *well-being* (*B* = −0.107, S.E. = 0.054, *p* = 0.048, β = −0.037), where coffee consumption was associated with more CUD symptoms and more CUD symptoms with lower well-being, and (3) e*nergy drink* → *CUD* → *well-being* (*B* = −0.098, S.E. = 0.049, *p* = 0.046, β = −0.034), where energy drink consumption was associated with more CUD symptoms and more CUD symptoms predicted lower well-being.

## Discussion

Our analyses, which focused on the relationship of self-reported ADHD symptoms and caffeine consumption showed some unexpected results. Caffeine consumption– whether treated as a continuous or a dichotomous variable, taking into account the type of the caffeinated beverage–did not correlate with the number of ADHD symptoms and was not different in the four ADHD groups. At the same time, ADHD symptoms showed a moderate positive association with the number of caffeine use disorder symptoms in the ANOVA and in both path models. Overall, these results suggest that it is not caffeine consumption *per se*, but rather the problematic use of caffeine that is related to self-reported ADHD symptoms. Looking at the differences between the four ADHD groups, the relationship seems to be linear: an increased probability of ADHD is associated with an increased number of caffeine use disorder symptoms. Caffeine consumption did not mediate the relationship between ADHD symptoms and well-being, but caffeine use disorder symptoms were a significant mediator in both path analyses with reduced well-being in participant with more caffeine use disorder symptoms. Together, these findings suggest that the hypothesis of (successful) self-medication does not apply to ADHD symptoms and caffeine consumption, but it seems that those who have more ADHD symptoms may be more prone to develop caffeine use disorder regardless of the magnitude of caffeine consumption. This result is partly contradicting and partly in line with the findings of Cipollone et al. (27): they found that caffeine consumption among soldiers with ADHD had a low, negative correlation with some of the ADHD symptoms, indicating successful self-medication attempts. On the other hand, they found a higher prevalence of SUD among soldiers with ADHD, which means that they probably have a higher vulnerability regarding the negative consequences of the use of certain substances. In the current study, lower well-being–which is associated with ADHD symptoms–is partly explained by the appearance of caffeine use disorder symptoms. It is possible that the relationship between the two disorders–ADHD and CUD–represents a common psychopathological factor (51) based on common environmental factors or a common genetic vulnerability [e.g., (52)]. It is also worth considering the type of caffeinated beverage: according to our results, tea consumption-although it is not associated with ADHD symptoms-has a positive association with perceived psychological well-being, which may confirm the recommendation of Liu et al. (9) that tea can be an appropriate agent for the treatment of adults with ADHD. Since coffee and tea are absorbed similarly leading to similar plasma-caffeine concentrations after either tea or coffee consumption [(53), cited by (54)], the difference in their psychological effects is probably not due to pharmacokinetics, but it rather originates from the different chemical composition or the different expectations associated with the two beverages. It is also possible that people who drink tea and those who drink coffee differ in certain physical and psychological characteristics (55, 56).

The consumption of coffee and energy drinks indirectly-through caffeine use disorder symptoms-and negatively contributed to psychological well-being. This is in line with our the previous results (36) indicating that a caffeine use disorder can indeed influence quality of life. Since lower well-being is probably influenced by factors other than ADHD and CUD as well (for example various physical illnesses, mental disorders), it is important to consider several other-potential confounding-variables in future studies.

Although we assumed certain relationships between ADHD symptoms, caffeine consumption, caffeine use disorder symptom and well-being, we could not present causal relationships because of the cross-sectional nature of the research. It is, therefore, possible that there is reverse or bidirectional causality between some of the variables. In addition, the sample was not representative of the Hungarian population: men, people with higher educational attainment and employment were overrepresented, which could affect caffeine consumption habits as well as ADHD symptoms since higher intellect can be a protective factor against the development and the negative consequences of ADHD (57). This divergence in demographics probably reflects the composition of the readership of the news website used for recruitment. Despite this distortion, we have achieved to reach a wide range of the population, as 444.hu is among the 25 most visited websites in Hungary. A further limit of the study is that we did not ask participants whether they have an ADHD diagnosis and a treatment history of ADHD, so the analysis was based only on the currently experienced ADHD symptoms. It is important to note that at least some of the symptoms should have occurred before the age of 12 for an ADHD diagnosis. However, in the current study 65.6% of the participants dated the first appearance of the ADHD symptoms at age of 13 or older, which may arise from the difficulty of recalling childhood memories, or suggest that the symptoms are not the signs of ADHD, but some other disorder (e.g., bipolar disorder, cluster B personality disorder). It is also possible, that several participants had late-onset ADHD symptoms, which may begin in adolescence or early adulthood (58) and which-according to some authors-can occur independently from childhood-onset ADHD (59). Including other relevant-potential confounding-variables, such as substance use other than caffeine, should be considered in future studies since they may affect the observed associations.

An important strength of the current study is that we included and examined ADHD symptoms, caffeine consumption, caffeine use disorder symptoms, and psychological well-being in a coherent and complex model, and also reflected on the differences between certain caffeinated products.

## Conclusion

The study found moderate associations between ADHD symptom severity, caffeine use disorder symptoms and psychological well-being: people with more ADHD symptoms had lower well-being, and caffeine use disorder symptoms partly mediated this relationship. Although the results indicate that people with more ADHD symptoms do not consume more caffeine in any form, but they are probably more sensitive for the reinforcing effects of caffeine, which lead to more CUD symptoms. Therefore, caffeine does not seem to be a compound for successful self-medication.

## Data Availability Statement

The raw data supporting the conclusions of this article will be made available by the authors, without undue reservation.

## Ethics Statement

The studies involving human participants were reviewed and approved by Research Ethics Committee of ELTE Faculty of Education and Psychology. The patients/participants provided their written informed consent to participate in this study.

## Author Contributions

CÁ: conceptualization, investigation, data curation, formal analysis, methodology, visualization, funding acquisition, writing–original draft, and writing–review and editing. RU and ZH: formal analysis and methodology. WB: writing–review and editing. ZD: conceptualization, investigation, methodology, funding acquisition, supervision, and writing–review and editing. All authors contributed to the article and approved the submitted version.

## Funding

This study was supported by the Hungarian National Research, Development and Innovation Office (Grant Numbers: KKP126835, K131635). Zsolt Horváth was supported by the ÚNKP-21-4 New National Excellence Program of the Ministry for Innovation and Technology from the source of the National Research, Development and Innovation Fund.

## Conflict of Interest

The authors declare that the research was conducted in the absence of any commercial or financial relationships that could be construed as a potential conflict of interest.

## Publisher's Note

All claims expressed in this article are solely those of the authors and do not necessarily represent those of their affiliated organizations, or those of the publisher, the editors and the reviewers. Any product that may be evaluated in this article, or claim that may be made by its manufacturer, is not guaranteed or endorsed by the publisher.
